# Label-free Imaging of Tissue Architecture during Axolotl Peripheral Nerve Regeneration in Comparison to Functional Recovery

**DOI:** 10.1038/s41598-019-49067-3

**Published:** 2019-09-02

**Authors:** Ortrud Uckermann, Joana Hirsch, Roberta Galli, Jonas Bendig, Robert Later, Edmund Koch, Gabriele Schackert, Gerald Steiner, Elly Tanaka, Matthias Kirsch

**Affiliations:** 1 Neurosurgery, Carl Gustav Carus University Hospital, TU Dresden, Dresden, Germany; 2 Clinical Sensoring and Monitoring, Department of Anesthesiology and Intensive Care Medicine, Faculty of Medicine, TU Dresden, Dresden, Germany; 3 CRTD/DFG-Center for Regenerative Therapies Dresden - Cluster of Excellence, Dresden, Germany

**Keywords:** Peripheral nervous system, Regeneration and repair in the nervous system, Translational research, Multiphoton microscopy

## Abstract

Human peripheral nerves hold the potential to regenerate after injuries; however, whether a successful axonal regrowth was achieved can be elucidated only months after injury by assessing function. The axolotl salamander is a regenerative model where nerves always regenerate quickly and fully after all types of injury. Here, de- and regeneration of the axolotl sciatic nerve were investigated in a single and double injury model by label-free multiphoton imaging in comparison to functional recovery. We used coherent anti-Stokes Raman scattering to visualize myelin fragmentation and axonal regeneration. The presence of axons at the lesion site corresponded to onset of functional recovery in both lesion models. In addition, we detected axonal regrowth later in the double injury model in agreement with a higher severity of injury. Moreover, endogenous two-photon excited fluorescence visualized macrophages and revealed a similar timecourse of inflammation in both injury models, which did not correlate with functional recovery. Finally, using the same techniques, axonal structure and status of myelin were visualized *in vivo* after sciatic nerve injury. Label-free imaging is a new experimental approach that provides mechanistic insights in animal models, with the potential to be used in the future for investigation of regeneration after nerve injuries in humans.

## Introduction

Peripheral nerve injuries are caused by trauma, degenerative diseases, repetitive compression or surgical interventions^1^. Consequences for the patient’s life are diverse, ranging from minor motor and sensory deficits to devastating functional disability. Nerve transections constitute a dramatic damage to the signal transducing neuronal cells. However, fortunately, peripheral nerves hold the potential for regeneration.

Peripheral neerve injuries are graded based on clinical and electrodiagnostic examinations according to the Seddon^2^ or Sunderland^3^ classifications. Surgical intervention is the therapeutic gold standard for severe injuries and aims at connecting the epineurium or perineurium of nerve stumps, leaving the axons and Schwann cells to respond to injury via their inherent biology^4^. Axons degenerate in the distal nerve stump while Schwann and immune cells provide a regenerative environment (Wallerian degeneration^5^). Denervated Schwann cells undergo a phenotype change toward non-myelinating cells. Consequently, they proliferate, gain phagocytic activity and remove myelin as well as cellular debris together with recruited resident and peripheral macrophages (for an overview see^6^). A successful nerve regeneration requires a preserved neuronal cell body, re-growing axons crossing the site of injury and reaching the distal nerve stump that acts as guidance structure and, finally, a correct target innervation. The success of any therapeutic intervention is assessed by the restoration of function, and the maximum outcome is not reached until months (Sunderland grade 1 nerve injury: 7 months; grade-2, -3, -4, and -5: 11, 14, 18, and 19 months, respectively^1^). The success of treatment is variable and complete repair and exact functional restorations are not possible despite advances in microsurgical technology and refinement of surgical techniques.

To date, there is a lack of technologies to track axonal regrowth, to monitor regeneration and to evaluate the healing process. Consequently, wait-and-see surgical decisions can lead to undesirable and less successful delayed repair procedures^7^ due to prolongation of the period of denervation. Identification of lacking intrinsic regeneration or failing treatment response would open the possibility to adjust therapeutic strategies and to improve patient’s functional outcome. Moreover, detailed monitoring of the regenerative response in animal models would allow better mechanistic insights as well as the development and evaluation of innovative therapies.

Label-free multiphoton microscopy probes the morphochemistry of the tissue and might be suitable to fulfill this demand. The combination of coherent anti-Stokes Raman Scattering (CARS) and endogenous two-photon excited fluorescence (TPEF) visualizes the intact and pathological nervous system and can be applied *in vivo*^8,9^ without induction of photodamage^10,11^. CARS is a non-linear variant of Raman spectroscopy and is usually applied to visualize the distribution of CH_x_ groups in the tissue. Therefore, it constitutes an excellent approach for the analysis of the structure of lipid-rich myelinated axonal sheaths^12^ and of intra- and extracellular lipid droplets^13^. CARS enabled to assess the micromorphology of the sciatic nerve in mouse model^14^ and to follow injury-induced changes of myelin in the rat sciatic nerve^15^. Endogenous TPEF shows neuronal cell bodies^16^ and activated inflammatory cells in spinal cord pathologies^17^. Myelin rearrangements and inflammation are hallmarks of nerve de- and regeneration.

Rodent models for sciatic nerve injury were intensively used in the last decades to elucidate the complex mechanisms underlying peripheral nerve regeneration in mammals^18^. However, sciatic lesions constitute a challenge to nerve regeneration in mammals. The regenerative response in rodents dramatically depends from species^19^ and strains^20^ and a full morphological and functional recovery is not always achieved for all injury models^21,22^. Moreover, sciatic injury in rodents frequently leads to a series of complications that make the animals suffer, hamper functional assessment and may lead to animal exclusion during the experiments^23^. While the use of mammal models has advantages towards a clinical translation, methodological uncertainty and experimental variability may challenge researchers^24^, hence there is interest towards alternative models. Here, the response after sciatic nerve injury was investigated in a highly regenerative model organism, the axolotl salamander *ambystoma mexicanum*. Urodele amphibians can regenerate peripheral and central nervous system and have been widely employed to study and understand the mechanisms of regeneration. The axolotl displays near complete functional recovery after severe sciatic nerve injury^25^. Therefore, it represents an ideal model to compare morphochemical findings with functional outcome.

Here, we analyzed the time course of de- and regeneration after sciatic nerve transection in the axolotl using label-free CARS/TPEF microscopy on cryosections and confirmed our findings *in vivo*. The morphochemistry of sciatic nerve was compared in two injury models with different kinetics of functional recovery. The aim was to determine key points of the regenerative process that may constitute the basis for successful functional outcome after peripheral nerve injuries.

## Results

### Morphochemistry of the intact nerve

**Figure 1:**
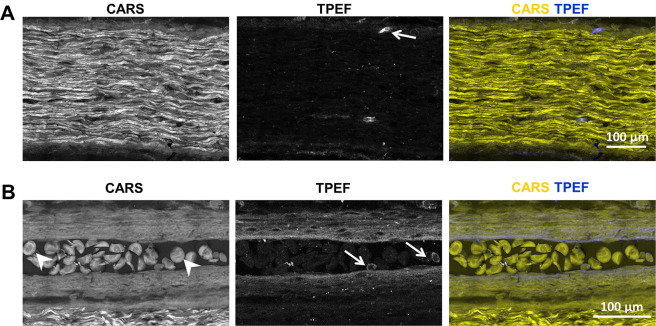
Intact sciatic nerve visualized using label-free multiphoton microscopy. **(A)** Intense CARS signal of aligned axons. Arrow indicates single cell with endogenous fluorescence. **(B)** Blood vessel adjacent to axonal tracts. Arrowheads indicate erythrocytes; arrows indicate blood cells with endogenous fluorescence. Single channel information for CARS and TPEF and overlay of CARS (yellow) and TPEF (blue).

Multimodal multiphoton imaging, i. e. the combination of CARS and TPEF, revealed the micromorphology of the intact axolotl sciatic nerve without the application of any labels or dyes (Fig. 1). Rehydration of cryosections before imaging restored the tissue morphology. Axonal myelin sheaths displayed an intense CARS signal (Fig. 1A) because of their high lipid content^12^. Fluorescent structures were rarely observed in cells at the nerve periphery (Fig. 1A, arrow). Furthermore, the technique allowed the visualization of the overall tissue structure and of cells. As an example, a blood vessel adjacent to axonal tracts is shown in Fig. 1B. The characteristic “fried egg” morphology of amphibian erythrocytes is depicted by CARS (Fig. 1B, arrowheads) while other blood cells were smaller and showed a fluorescent cytoplasm (Fig. 1B, arrows).

### Nerve regeneration and time course of functional recovery

**Figure 2:**
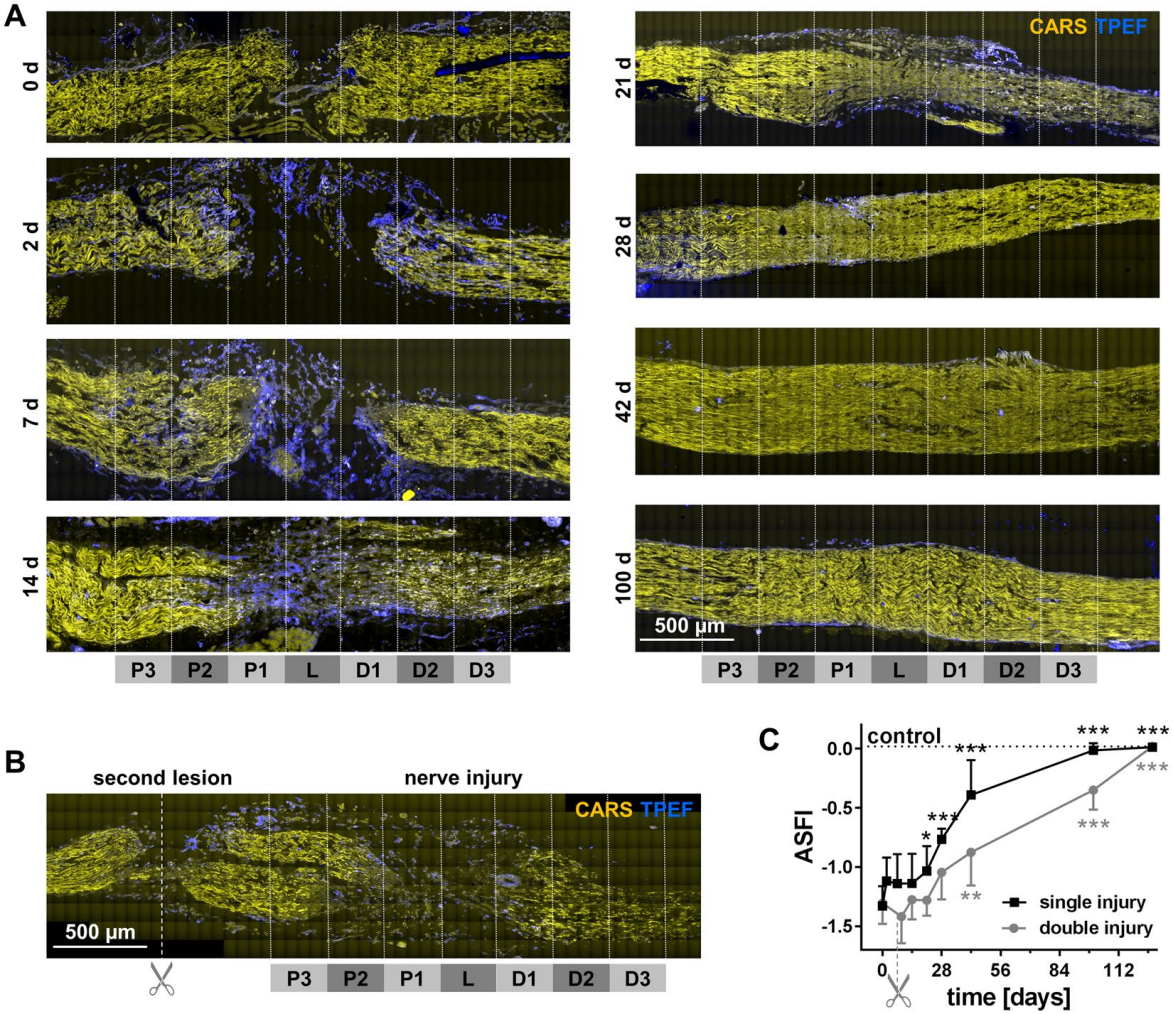
Label-free multiphoton microscopy of the axolotl sciatic nerve and functional outcome after injury. (**A**) CARS/TPEF-images of sciatic nerve longitudinal sections at different time points after nerve injury (single injury model) **(B)** CARS/TPEF image of the sciatic nerve nine days after injury and two days after second lesioning (double injury model). The regions for quantitative analysis are indicated in A and B: P3-1: proximal 3-1; L: lesion; D1-3: distal 1-3. (**C)** Axolotl sciatic nerve functional index (ASFI) for both injury models. Dotted line indicates ASFI of animals without injury (control). Asterisks indicate significant differences to the ASFI at 0d, i. e. directly after injury (*P < 0.05, **P < 0.01, ***P < 0.001; one way ANOVA followed by Bonferroni’s multiple comparisons test).

CARS/TPEF imaging was performed at different time points after transection of the axolotl sciatic nerve (day 0 to day 100, Fig. 2A) and referenced with standard immunohistochemistry (Supplementary Figs S1 and S2). On the day of transection, the two stumps of the nerve were clearly separated by a gap of 200–300 µm. The comparison with the morphology of the uninjured nerve (Fig. 1A) shows, that the structure of the nerve remained largely unchanged. Cells began to populate the space between the two stumps two days after injury. This area was characterized by a pronounced endogenous fluorescence until day 14 after injury. At day 21, tissue reconnection was observed. The multiphoton images reveal the presence of a tissue bridge lacking any notable fluorescence. Then, the overall tissue structure further improved and the CARS signal intensity increased gradually. At day 100 after injury, the entire nerve displayed a homogenous macromorphology comparable with the uninjured nerve (compare Fig. 1A).

To interfere with the process of successful regeneration, we introduced a second lesion proximal to the first one seven days after initial nerve injury. The additional transection of axons strongly affected the tissue morphology in regions proximal to the initial site of injury (Fig. 2B).

The phenotype of peripheral nerve injury is easily accessible and was evaluated in both models: The range of movement of knee and ankle as well as the maximal toe spread of the injured leg were monitored and employed to calculate the axolotl sciatic nerve functional index (ASFI, Fig. 2C). In the first two weeks after nerve transection, functional performance was poor, consistent with the presence of a gap between the nerve stumps. The onset of functional recovery was observt at day 21 after injury which is the time point when tissue reconnection was found (compare Fig. 2A): The ASFI was significantly higher than at the day of injury (P < 0.05, one way ANOVA followed by Bonferroni’s multiple comparisons test). The ASFI further increased during regeneration. No difference to the ASFI before injury was detectable after 100 days. The time course of functional improvement was different in the double injury model. The ASFI was significantly lower than after single injury at multiple time points (28, 42 and 100 days after initial injury), indicating a delayed functional regeneration in the double injury model (P < 0.05, P < 0.001 and P < 0.01, one way ANOVA followed by Bonferroni’s multiple comparisons test). Moreover, the onset of functional recovery and full functional recovery were detected later, namely at day 42 and 128 after injury, respectively.

To evaluate the relationship of tissue regeneration and functional improvement, the tissue morphochemistry was investigated by quantitative analysis of CARS and TPEF signals in regions proximal, distal and within the lesion.

### Analysis of the CARS signal

**Figure 3:**
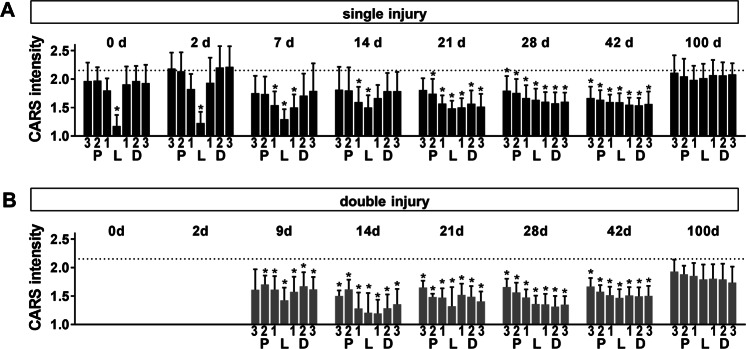
CARS signal intensity after sciatic nerve injury. Normalized CARS signal intensity in regions proximal to the lesion (P 3-1), distal to the lesion (D 1-3) and within the lesion (L) as indicated in Fig. 2A/B at different time points after initial injury of the sciatic nerve. (**A)** single injury model **(B)** double injury model. Bars show mean and SD. Dotted line indicates CARS intensity of intact control nerve. Asterisks indicate significant differences vs. control (P < 0.05, one way ANOVA followed by Bonferroni’s multiple comparisons test).

Figure 3A shows the normalized CARS signal intensity along the nerve in the single injury model at different time points after transection.

On the day of injury, the CARS signal intensity in the lesion was significantly reduced and reached almost background values. This was in accordance with the presence of a gap between the nerve stumps. Afterwards, the CARS signal intensity of adjacent regions gradually decreased and was significantly lower seven days after injury. The CARS signal intensity was reduced in all proximal and distal regions at day 28 and day 42 after injury and this reduction was more pronounced in tissue distal to the lesion site. The CARS signal intensity at the position of the lesion started to increase again after seven days and was similar to the CARS signal intensity in distal and proximal regions 28 days after injury. Control values were reached in all regions 100 days after injury. Similar changes were found in the double injury model (Fig. 3B). However, the CARS signal intensity was significantly reduced in all peripheral and distal regions already at day 14 and a general trend towards lower CARS signal intensities was observed at all time points.

**Figure 4:**
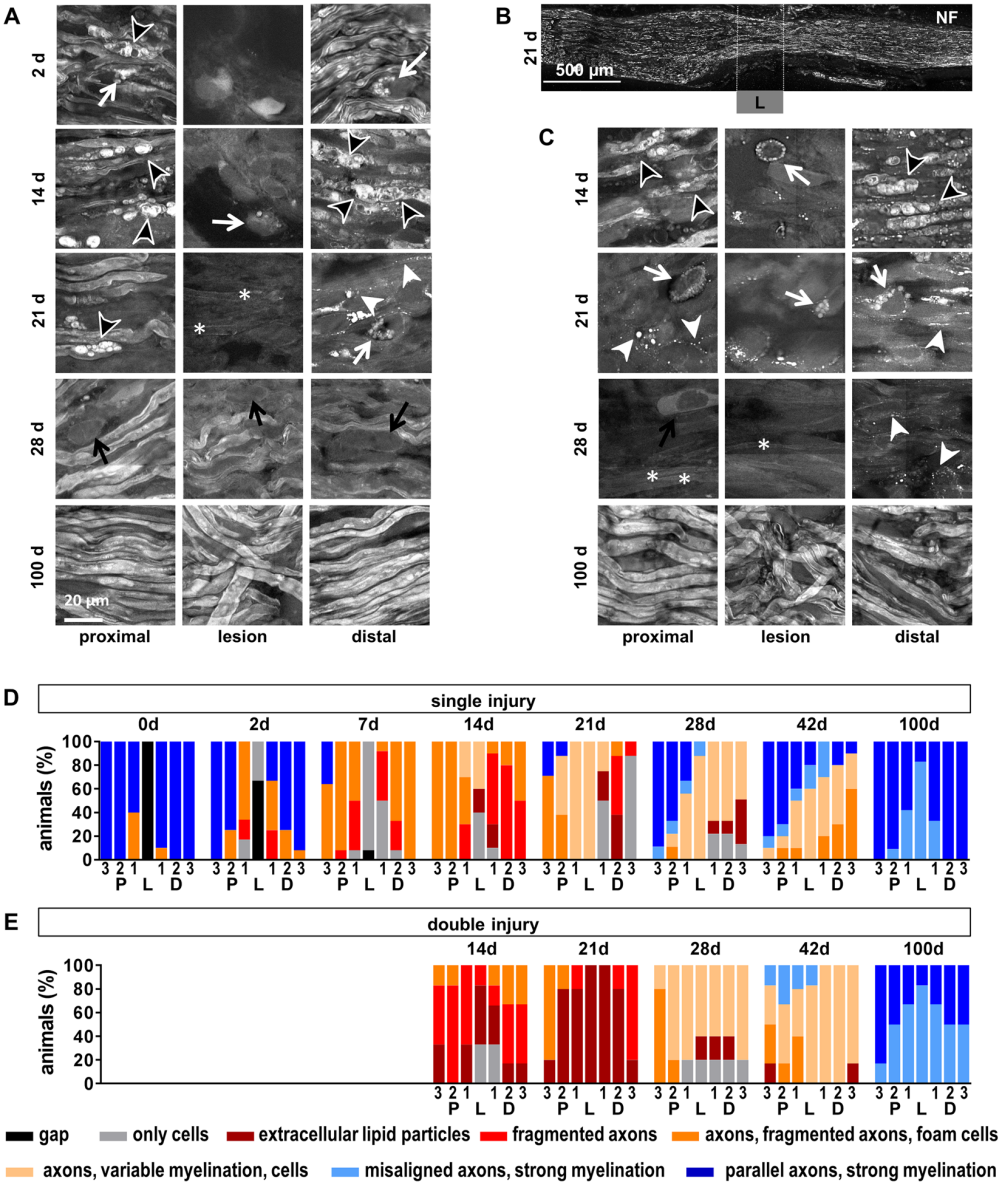
Micromorphology of the regenerating nerve shown by CARS. (**A)** CARS images at different time points after sciatic nerve dissection as indicated for single injury model. Representative examples proximal to the lesion, at the lesion and distal to the lesion. (**B)** Reference immunohistochemistry for neurofilament H of the axolotl sciatic nerve 21 d after injury. The region of the lesion is indicated (L) **C:** CARS images at different time points after sciatic nerve dissection as indicated for double injury model. White arrows: Cells with intracellular lipid droplets, black arrows: Cells, white arrowheads: Extracellular lipid droplets, black arrowheads: Ovoids, asterisk: Not/weakly myelinated axons. (**D/E)** Semi quantitative analysis of the micromorphology based on CARS imaging in the single (**D**) and double (**E**) injury model using a scoring system. Quantification was performed in seven regions along the nerve (P3-1, L, D1-3, indicated in Fig. 2A/B). The bars indicate the percentage of samples that fall in the given categories.

Changes in the CARS signal intensity might be attributed to changes in axonal myelin, tissue architecture or accumulation of extracellular lipid particles representing myelin debris and intracellular lipid droplets. Therefore, we analyzed the morphochemical changes after sciatic nerve transection in more detail. Figures 4A,C show representative examples of sciatic nerve micromorphology in the single and double injury model, respectively, and illustrate the differences in regions proximal, inside and distal to the lesion. Based on the CARS signal, the micromorphology of the sciatic nerve was evaluated using a scoring system in both models (Fig. 4D,E).

Immediately after injury, the two nerve stumps displayed an intact myelin structure. However, myelin damage was confirmed in regions adjacent to the lesion in a subset of animals. Two days after injury, cells were observed at the location of the lesion in one third of the animals. Lipid-laden foam cells (Fig. 4A, white arrows) and myelin ovoids (Fig. 4A, black arrowheads) were detected in adjacent regions and myelin fragmentation started. Seven days after injury, these signs of degeneration were found in more distant regions as well, and the CARS signal intensity of axonal myelin was reduced. The former lesion was filled with cells in almost all animals at this time point.

Two weeks after injury, the situation remained unchanged in regions proximal to the lesion. At the position of the lesion, sparse axons with weak or no myelin were found in 40% of the animals, while the lesion was filled by cells including foam cells in the rest of the animals. In 20% of animals, extracellular lipid particles were observed. Massive fragmentation of myelin was found and foam cells were detected in distal regions.

One week later (21d), parallel, myelinated axons resembling those in the intact nerve were observed in proximal regions distant to the site of injury in a few animals. However, in the majority of animals, proximal nerve micromorphology was characterized by a combination of continuous and fragmented axons. Foam cells were observed in some cases. The CARS signal intensity of myelin sheaths was higher compared to previous time points (see examples in Fig. 4A). Continuous axonal structures were found in all animals at the position of the former lesion, in coincidence with the onset of functional recovery (compare Fig. 2C). However, a strong variability in axonal density and CARS signal intensity of myelin has to be taken into account. The presence of axonal structures at the former lesion site was confirmed by neurofilament immunohistochemistry (Fig. 4B).

Distal to the lesion, myelin fragmentation and degradation proceeded, resulting in a morphology dominated by cells and lacking axonal structures. Extracellular lipid particles and myelin fragments were rarely observed in some animals.

Four weeks after injury, parallel and myelinated axons as well as axons displaying variable degrees of myelination in combination with cells (Fig. 4A, black arrows) were found in proximal regions. Inside the lesion, strongly myelinated axons, although being misaligned, were detected in one animal. Distal to the lesion, more animals displayed axonal structures and the evidence of degenerative findings (namely lack of axonal structures and presence of extracellular lipid particles) was reduced compared to the previous time point. In continuation of this trend, more animals displayed an intact axonal structure and less degeneration was found at day 42, being consistent with the idea of regrowth of proximal axonal structures that bridge the lesion and repopulate distal regions of the nerve. One hundred days after nerve transection, axons with myelin sheaths displaying an intense CARS signal intensity were found in all animals. However, the alignment of axonal fibers was impaired within the former lesion site and in adjacent areas.

The overall micromorphology in the double injury model resembled the micromorphological characteristics of regions distal to the lesion in the single injury model. Furthermore, the restoration of regular axonal structures was delayed (Fig. 4C,E).

Two weeks after transection of the sciatic nerve (and 7 d after induction of the second lesion), fragmentation of myelin was observed in proximal and distal regions. In addition, extracellular lipid particles were detected in approximately one quarter of the animals. Axonal structures were rarely observed. Cells were located in the lesion and extracellular lipid particles were sometimes observed. Myelin degradation proceeded and only a few fragments of myelin were detected 21 d after injury. The nerve’s appearance was dominated by the presence of cells including foam cells, and extracellular particles were found in all regions. Though, continuous axonal structures in combination with myelin fragments and foam cells were found in most animals proximal to the lesion at distances larger than 600 µm. One week later (28 d), axonal structures, even if displaying no or weak myelination, were found in all regions, however not in all animals. The presence of lipid particles was restricted to the former lesion and distal regions. After 42 d, axonal structures were found in virtually all animals. Moreover, myelin sheaths characterized by intense CARS signal intensity were found in regions proximal to the injury in some animals. Strongly myelinated axons were observed in all regions 100 d after injury. However, parallel alignment was limited to less than half of the animals investigated.

The comparison of the micromorphology shown by CARS (Fig. 4) to the functional improvements of the two models (Fig. 2C) reveals a significant increase in ASFI at time the time point when axonal structures within the lesion were observed in all animals investigated. Furthermore, the initial delay in the double injury model was not compensated but seemed to propagate to later time points.

### Analysis of the TPEF signal

**Figure 5:**
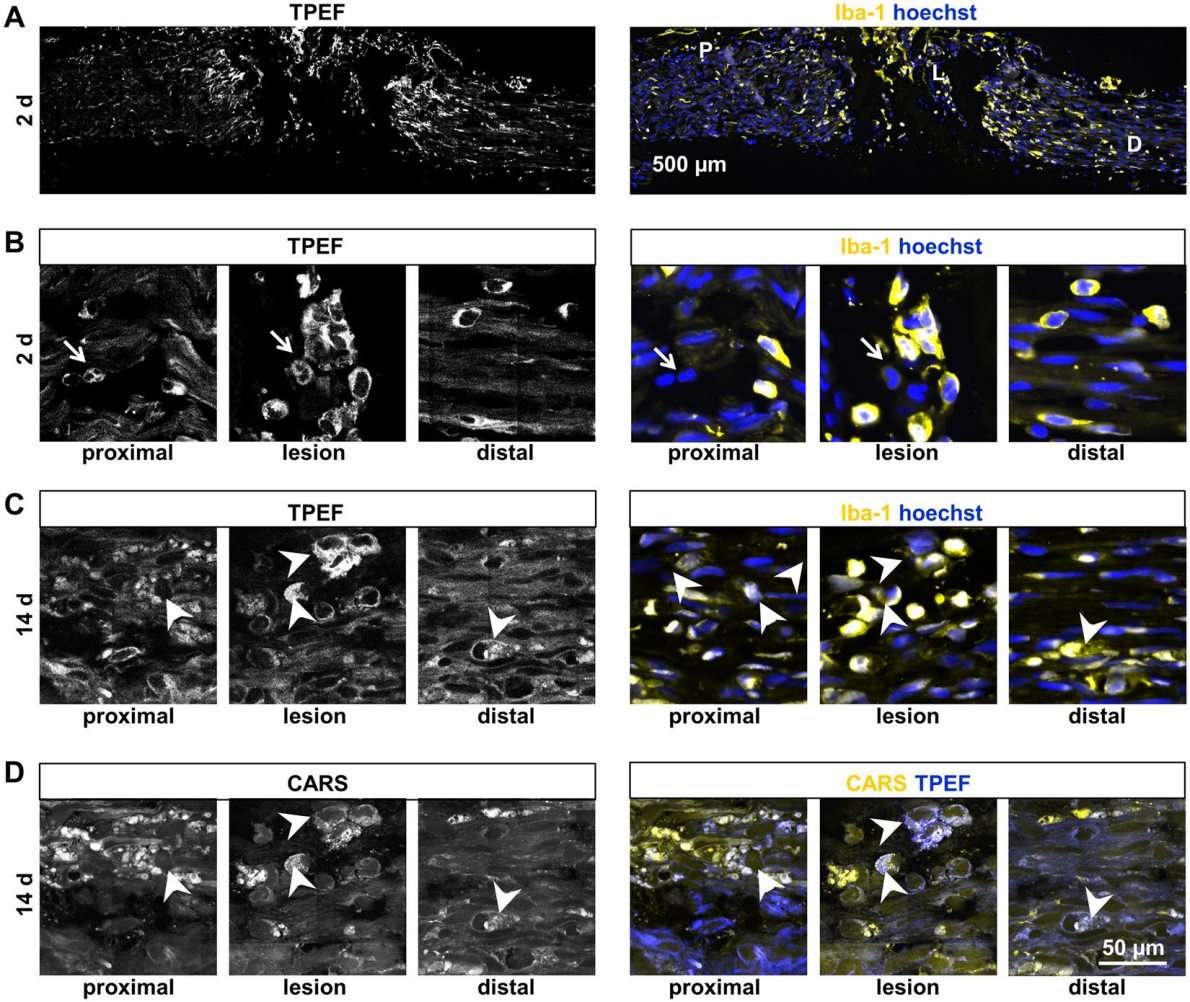
Endogenous TPEF signal. Immunohistochemical staining for the macrophages marker Iba-1 in comparison to TPEF (**A)** Overview image (same sample as shown in Fig. 2) two days after sciatic nerve dissection. Magnifications of the position indicated are shown in (**B)** (P: proximal, L: lesion, D: distal) (**B/C)** Cellular comparison of endogenous TPEF and Iba-1 immunohistochemistry proximal, distal and within the lesion 2 d and 14 d after injury (**D)** CARS channel and composite images of CARS and TPEF of the areas shown in **C**. arrows: Iba-1-negative fluorescent cells; arrowheads: cells with fluorescent cytoplasmic inclusions.

The overview images (Fig. 2) clearly revealed that sciatic nerve injury induced fundamental changes in the pattern of the tissue’s endogenous fluorescence. The comparison of the TPEF-overview image with the reference Iba-1 immunohistochemistry for macrophages showed similar patterns of both signals two days after nerve transection (Fig. 5A).

At higher magnifications, it became clear that the TPEF signal originated from a subpopulation of cells within the sciatic nerve and that the fluorescence was confined to the cytoplasmic compartment (Fig. 5B). Two days after transection, we found cellular matching of the TPEF signal and Iba-1 immunofluorescence in regions proximal and distal to the lesion and within the cellular bridge that was forming at the lesion site. Almost all cells showing endogenous fluorescence were positive for Iba-1 and, therefore, represented macrophages. Exceptions, i.e. fluorescent cells that were Iba-1 negative, might represent mitotic cells based on shape and Hoechst staining (Fig. 5B, arrows). This is supported by previous studies: Their cellular morphology and localization resembles the macrophages that were identified in the axolotl forearm flexor nerve after injury^26^ and activated microglia/macrophages were likewise shown to exhibit endogenous fluorescence in the injured and pathologic rodent spinal cord^17,27^.

At later time points after nerve transection, the majority of Iba-1 positive cells still had a counterpart in the TPEF channel. However, they displayed variable intensities of endogenous fluorescence. Figure 5C shows examples 14 d after injury and illustrates that the initial one-to-one correlation of cells with endogenous TPEF and positive Iba-1-immunohistochemistry was lost. Cells with fluorescent cytoplasmic inclusions were detected in all regions analyzed (arrowheads in Fig. 5C). Those fluorescent inclusions displayed an increased CARS signal and might, therefore, represent droplets of (oxidized) lipids (Fig. 5D).

Our findings are consistent with the hypothesis that the chemical origin of endogenous fluorescence might be fluorescent cytoplasmic pyridinic coenzymes that are involved in the oxidative burst. Phagocytosis of myelin induced oxidative burst in microglia within 30 min^28^, synchronizing the macrophage population. We hypothesize, that this synchronization is lost and that there are macrophages in different stages of maturation and/or with different functions at later time points. Cells full of lipid droplets and lacking endogenous fluorescence might represent macrophages that stopped phagocytosis and production of endogenous fluorophores or autophagic Schwann cells^29,30^.

**Figure 6:**
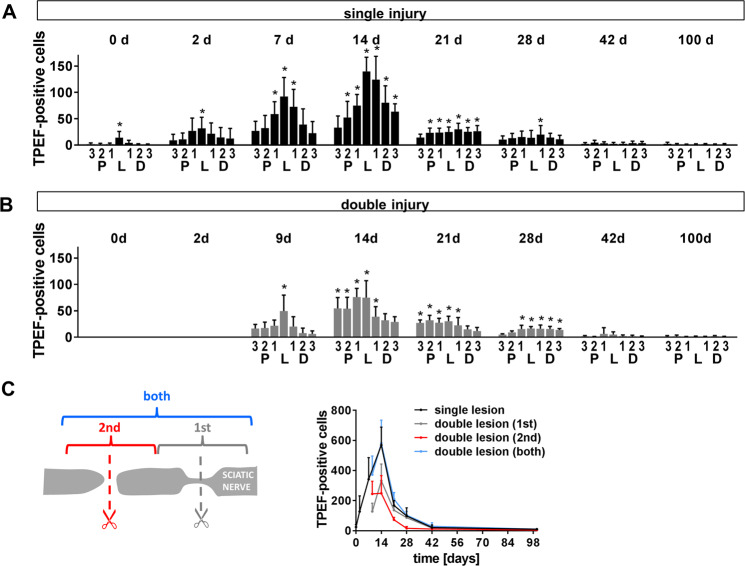
Changes in TPEF-positive cells after sciatic nerve injury. (**A)** Number of TPEF-positive cells in regions proximal to the lesion (P3, P2, P1), distal to the lesion (D1, D2, D3) and within the lesion (L) at different time points after initial injury of the sciatic nerve. (**A)** single injury model **(B)** double injury model. Bars show mean and SD. Asterisks indicate significant differences vs. control (intact nerve). (**C)** Total number of TPEF positive cells (sum of all regions investigated) at different time points after nerve transection (mean and SD).

The number of TPEF-positive cells is shown in Fig. 6A for the single injury model. At 0 d, i.e. ~8 h after transection, it was significantly elevated to 14.1 ± 11.8 (mean ± SD) inside the lesion compared to control values of 1 ± 0.2. The number of TPEF-positive cells inside the lesion strongly increased until day 14 (139.9 ± 26.9) and declined afterwards. Pre-injury low numbers of TPEF-positive cells were found 42 d after injury (2.2 ± 2.6). The increase in TPEF-positive cells was less pronounced in regions proximal and distal to the lesion, but followed the same time course. Similar numbers of TPEF-positive cells were observed in all regions along the nerve 21 d after nerve transection and at later time points.

Figure 6B illustrates the distribution of TPEF-positive cells along the nerve in the double injury model. The time course was similar to the one after single injury with maximal numbers at the lesion site after 14 d. However, this maximal number of fluorescent cells at 14 d (75 ± 32.1) was considerably lower than in the single injury model, probably because of the recruitment of cells to the second lesion. The comparison of the total number of TPEF-fluorescent cells in the single injury model with the one in the double injury model at both lesion sites indicated similar numbers of TPEF-positive cells in both models at all time points (Fig. 6C).

Comparing the inflammatory response revealed by TPEF-positive cells to the functional recovery (Fig. 2C) does not suggest a clear relationship. While onset and completion of functional recovery was observed at different time points in both models, the time course of inflammation was similar (Fig. 6C).

### *In vivo* monitoring

**Figure 7:**
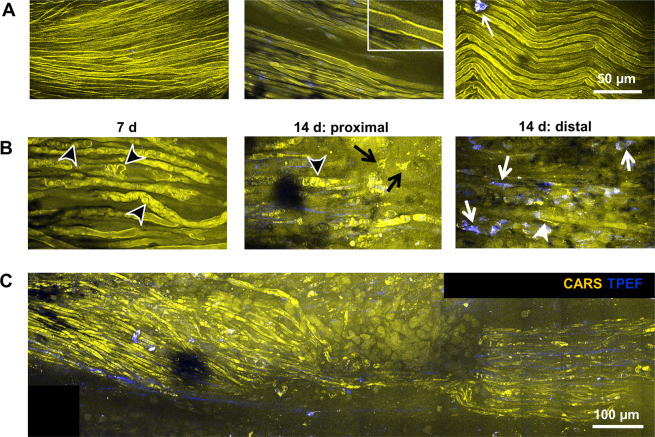
*In vivo* multiphoton imaging. (**A)** Intact sciatic nerve. Inset shows a Schmidt-Lanterman incisure **(B)** Examples of sciatic nerve micromorphology 7 d and 14 d after transection. (**C)** Overview image of the sciatic nerve 14 d after transection reconstructed from Z-stack. Composite images of CARS (yellow) and TPEF (blue). White arrows: TPEF-positive cells; black arrowheads: ovoids; black arrows: foam cells; white arrowheads: extracellular lipids.

Multiphoton imaging of the exposed sciatic nerve was tested *in vivo*. The intact sciatic nerve (Fig. 7A) was characterized by aligned, parallel axons with myelin sheaths that displayed an intense CARS signal. This morphochemistry was in agreement with the data obtained on cryosections (compare Fig. 1A). Moreover, the technology allowed the visualization of Schmidt-Lanterman incisures (Fig. 7A, inset). However, a wavy appearance of axonal alignment was occasionally observed. Fluorescent structures or cells were rarely detected (white arrow in Fig. 7A). The injury-induced changes in axonal myelin described using cryosections were likewise observed under *in vivo* conditions: Seven days after injury, the presence of ovoids indicated myelin breakdown (Fig. 7B, black arrowheads). Two weeks after injury, nerve degeneration was still characterized by myelin degradation with ovoid formation and axonal fragmentation. Moreover, cells with intracellular lipid droplets (black arrows), extracellular lipid particles (white arrowheads) and an increased number of TPEF-positive cells (white arrows) were observed (Fig. 7B). The endogenous TPEF signal was slightly different from the pattern detected on cryosections. The density of TPEF-positive cells was not as high as expected, and fluorescent fibrous structures were found that were not seen in images of cryosections. However, the overall structure of the nerve, the location of the lesion and invading cells as well as single axonal structures and the status of myelination could be assessed *in vivo* using label-free multiphoton microscopy (Fig. 7C).

## Discussion

Label-free multiphoton imaging revealed tissue microstructure in two models of peripheral nerve injury with different time course of functional recovery. The analysis of the endogenous TPEF predominantly shows the extent of the inflammatory response while CARS imaging allowed the assessment of the overall tissue architecture, of axonal myelin and formation of lipid droplets. We confirmed that i) label-free multiphoton microscopy reveals the sequence of tissue de- and regeneration, ii) axonal regrowth shown by CARS is related to functional recovery while inflammation shown by TPEF is not; and iii) the technology is able to provide this information *in situ*.

The sequence of events visualized by CARS and TPEF is largely consistent with previous studies on vertebrate sciatic nerve injury. The invasion of macrophages, morphology of cells and cytoplasmic accumulation of lipid droplets were in accordance to studies of the degenerating rat peripheral nerve using conventional label-based techniques^5,31,32^. Wallerian degeneration of the distal nerve stump was clearly resolved, followed by the remyelination of newly formed axons. In the rat model, downregulation of myelin expression and ovoid formation was observed within two days after sciatic nerve transection (for review see^33^). The micromorphology observed in the transected axolotl nerve by CARS was similar to that seen in the crushed rat nerve^15^. However, not all events of the complex sequence of sciatic nerve regeneration^18^ could be addressed by label-free imaging.

Recently, the mouse sciatic nerve was analyzed by visualization of myelin using stimulated Raman scattering (SRS) imaging addressing CH_x_. The presence of ovoids was identified as early morphological marker for nerve degeneration comparable to the initial observation of muscle denervation by electromyography and before impairment of motor nerve function in an ALS model^34^. In addition to myelin changes that were likewise visualized by SRS imaging, we were able to observe non/weakly myelinated axons. This might be explained by the fact that CARS provides additional non-resonant information that can be used to monitor morphological tissue structure besides the resonant, chemically specific signal related to vibrations of CH_x_-groups^35^. This morphological component of the CARS-signal might be exploited for visualization of structures lacking chemical contrast in the *in vivo* setting, thus expanding the information about the status of regeneration and might offer an advantage compared to other label-free imaging technologies like SRS. However, SRS can be used to address proteins in addition to lipids and could, therefore, theoretically enable to address not myelinated axons as well.

We found differences in the time course of regeneration between the single and the double injury model tested in this study. In particular, the successful bridging of the site of injury by axons was not yet achieved in all animals after double injury before 42 d. Likewise, the onset of functional recovery was delayed. Interestingly, the inflammatory response was not time shifted – there was only a spatial redistribution of cells. This is in accordance with the conclusion of earlier research^36^, showing limited correlation of the inflammatory changes after peripheral nerve injury and the speed of axonal regeneration. Moreover, the proliferative Schwann cell response to nerve injury was found to be independent of macrophage-derived signaling^37^.

In both injury models, the onset of functional recovery was observed at the time point when the presence of axonal structures within the former lesion site was confirmed in all animals. It is well known that functional regeneration after nerve injuries depends on successful bridging of the defect, which means that outgrowing neurites have to reach the distal nerve stump. This step determines the final result and the fate of nerve injury^38^. Therefore, axonal structures at the position of the former lesion that were detected by CARS are important for functional recovery. Moreover, the extent of myelination, which can be assessed by CARS, might be directly linked to axonal function. For instance, myelin sheaths of regenerated axons (dog / rat) are thinner than those of uninjured control^39^ and less myelin may be linked to lower nerve conduction velocity. The therapeutic improvement of axonal diameter and thickness of myelin sheaths resulted in higher conduction velocity in the regenerating rat sciatic nerve^40^. Furthermore, a correlation of conduction velocity to increased axon diameter and myelin thickness was observed in sciatic nerves in postnatal development of the rat^41^.

CARS imaging does not allow a prediction about reinnervation of the target which is usually compromised after nerve transection and contributes to impaired functional outcome^42^. From a clinical point of view, the readout of target innervation is the functional outcome and well accessible, however, without the possibility to therapeutically promote nerve regeneration any more. CARS imaging could provide evidence for lacking regeneration or the amount of axons and the extent of myelination at early time points. It thereby could reveal functionally relevant events at the former injury site when therapeutic interventions are possible and personalized treatment may result in improved functional outcome of the patient. Successful therapeutic monitoring using the label-free optical method SRS and addressing myelin has been already proven in the mouse model after peripheral nerve injury^34^. In this study, repetitive *in vivo* imaging of the injured sciatic nerve was performed without any damage. Technically, the application of label-free multiphoton imaging as clinical monitoring tool is challenging and requires further engineering development. There are rapid technical advances in the field of laser sources for biomedical applications^43^ and the development of special laser sources for excitation of the CARS process^44,45^ will facilitate future biomedical exploitation. Commercial CE-certified medical CARS imaging systems are already available^46^. Furthermore, CARS endoscopic solutions are currently beeing developed^47–49^. Inherently, any optical based bioanalytical imaging tool is limited to the field of view and penetration depth in tissue is small. Therefore, the monitoring of neural structures will require introduction of the optical probe in close approximation to the structure of interest. In a clinical context, this might be more preferable than delaying treatment decisions and missing early time points for therapeutic intervention.

Label-free multiphoton imaging opens the possibility for diagnostic analysis and therapeutic monitoring of nerve injuries, which do not exist so far. In a first step, label-free multiphoton imaging could be applied during surgery for nerve reconstruction. Here, the injured nerve is exposed and optically accessible, so that the technique could provide an optical, non-destructive biopsy to analyze the extent and the type of nerve injury. The visualization of the damage, the integrity of axonal tracts and of the progress of spontaneous regeneration could help to properly grade injuries and potentially guide surgical treatment decisions^50^.

## Materials and Methods

### Ethics statement

All animal experiments were performed in accordance with the guidelines of the Technische Universität Dresden, based on national laws that are in full agreement with the European Union directive on animal experimentation. They were approved by the Regional Council (Landesdirektion Sachsen, Germany, AZ: DD24-5131/338/43).

### Animal experiments

Axolotl salamanders (Ambystoma mexicanum) were bred in the CRTD/DFG-Center for Regenerative Therapies Dresden. Adult animals of 12 cm size were maintained in individual tanks filled with tap water at 18–20 °C in a 12:12 light-dark cycle^51^. Animals were anesthetized in a solution containing 0.03% (wt/wt) ethyl-p-aminobenzoate (Sigma Aldrich, St. Louis, Missouri, USA). Transection of the sciatic nerve was performed as described elsewhere^25^. Briefly, a 1 cm incision was created and the skin and musculature were gently retracted. The sciatic nerve was exposed and transected at the point of branching of the neighboring vein. The site of injury was labeled with a surgical thread. Wounds were closed and animals were allowed to recover from anesthesia to perform functional testing (see below).

### Axolotl sciatic functional index (ASFI)

Functional testing was performed as described elsewhere^25^. Animals were placed in a swim tank with continuous unidirectional water flow. A digital video camera was used to monitor the swimming axolotl including the hindlimbs. The maximum range of movements of the hip, the knee, and the ankle as well as the distance of toe spread were analyzed in single frames. Values for the injured leg were normalized to those of the contralateral (not injured) side.

### Tissue processing

Animals were sacrificed by an overdose of the anesthetic drug 0, 2, 7, 14, 21, 28, 42, 100 or 128 days after injury. The sciatic nerve was isolated, fixed overnight in 10% formalin in 10% Minimum Essential Medium (Sigma Aldrich) and cryoprotected in sucrose (30% for 24 h), embedded in tissue freezing medium (Leica, Nussloch, Germany) and frozen on dry ice. Finally, 12 µm thick longitudinal cryosections were prepared on glass slides and stored at −20 °C until use.

### Multimodal multiphoton microscopy

The multiphoton microscope used was described elsewhere^11^. The upright Axio Examiner Z.1 with scanning module LSM 7 (Carl Zeiss AG, Jena, Germany) was equipped with two Erbium fiber lasers: Femto Fiber pro NIR (781 nm, pulse length of 1.2 ps, maximum nominal power 100 mW) and Femto Fiber pro TNIR (set to 1005 nm to resonantly excite the symmetric stretching vibration of methylene groups at 2850 cm^−1^, pulse length of 0.8 ps, emitted power 1.5 mW; both Toptica Photonics AG, Gräfelfing, Germany). A C-Apochromat 32x/0.85 objective was used. CARS and TPEF were simultaneously excited and acquired. The endogenous TPEF signal was acquired in reflection (band pass 500–550 nm). The CARS signal was acquired in transmission (on cryosections) and in reflection (*in vivo*) with a band pass filter 633–647 nm.

Unstained cryosections were rehydrated with phosphate buffered saline and coverslipped before imaging.

### *In vivo* imaging

*In vivo* imaging was demonstrated in two animals at two time points each (7 d and 14 d after injury). Exposure of the sciatic nerve was essentially performed as above under ethyl-p-aminobenzoate anesthesia carefully avoiding any damage to the neural structures. The animal was positioned under the microscope, covered with wet paper towels and imaging was performed in reflection mode. Afterwards, the wound edges were compressed and closed without any further surgical measures. The animals were allowed to recover in their basins.

### Immunohistochemistry

For immunohistochemistry, the tissue sections were washed three times in 0.3% PBS-TWEEN, treated with citrate buffer (pH 6.0, Sigma-Aldrich) at 90 °C for 15 min, blocked with 3% normal goat serum for 20 min and probed with the primary antibody for 1 h. After washing with 0.3% PBS-TWEEN, the sections were incubated with the secondary antibody for one hour followed by further washing steps and staining of nuclei using Hoechst (Hoechst Stain solution, Sigma-Adrich). The following combinations were used: Rabbit anti-Iba-1 (1:200, Wako Pure Chemical Industries, Osaka, Japan) and Neurofilament-H (NF, 1:200, Merck Millipore, Billerica, Massachusetts, USA) were detected using goat anti-rabbit Alexa Fluor555 (1:200, Life technologies, Carlsbad, California, USA). Rat anti Myelin Basic Protein (MBP, 1:50, Genetex International Corporation, Hsinchu City, China) was detected using goat anti-rat Alexa Fluor488 (1:200, Life technologies). βIII tubulin (1:100, R&D Systems, Minneapolis, Minnesota, USA) was visualized using goat anti-mouse Alexa Fluor488 (1:200, Life technologies).

### Multiphoton image analysis

Analysis was performed on unprocessed single channel images. The site of nerve injury was localized based on the position of the surgical thread. Analyses were performed in seven adjacent regions of interest (300 µm width) that were defined along the nerve. Three were located proximal to the site of injury (P3, P2, P1), one was located at the site of injury (L), and three were located distal to the site of injury (D1, D2, D3).

The signal intensity of the CARS channel was measured using Fiji software^52^. The intensity values in the regions of interest were normalized to the CARS intensity of the background that was measured on every image in a position without tissue to compensate for differences in laser power or alignment that might vary among different days of acquisition.

The morphological characteristics of the CARS signals were analyzed by visual inspection of CARS images. Each region of interest was assigned to one of eight categories: 1. Presence of parallel axons with strong myelination, no ovoids, no/rare cells, appearance like the intact control nerve; 2. Misaligned axons with strong myelination, no ovoids, no/rare cells; 3. Axons with variable degrees of myelination ranging from none/weak to strong and obvious presence of cells; 4. Presence of continuous or fragmented axons, fragments of myelin and/or ovoids and/or foam cells; 5. Only fragmented axons, with variable degrees of myelination, with or without foam cells; 6. Presence of extracellular lipid particles, presence of cells, with or without foam cells, with or without isolated myelin fragments, absence of axons; 7. Presence of cells, absence of axons, absence of myelin fragments, absence of lipid droplets; 8. Gap, absence of tissue structures.

TPEF-positive cells were counted manually, thresholding for background fluorescence was performed according to^17,53^.

## Supplementary information

Supplemantary Material

## Data Availability

The data that support the findings of this study are available from the corresponding author upon reasonable request.
