# Author Correction: Label-Free Imaging of Tissue Architecture during Axolotl Peripheral Nerve Regeneration in Comparison to Functional Recovery

**DOI:** 10.1038/s41598-020-72252-8

**Published:** 2020-09-15

**Authors:** Ortrud Uckermann, Joana Hirsch, Roberta Galli, Jonas Bendig, Robert Later, Edmund Koch, Gabriele Schackert, Gerald Steiner, Elly Tanaka, Matthias Kirsch

**Affiliations:** 1 Neurosurgery, Carl Gustav Carus University Hospital, TU Dresden, Dresden, Germany; 2 Clinical Sensoring and Monitoring, Department of Anesthesiology and Intensive Care Medicine, Faculty of Medicine, TU Dresden, Dresden, Germany; 3 CRTD/DFG-Center for Regenerative Therapies Dresden - Cluster of Excellence, Dresden, Germany

Correction to: *Scientific Reports* 10.1038/s41598-019-49067-3, published online 02 September 2019

The information in this Article is incomplete. The number of animals used for each experiment was not provided. Updated figures, in which the n values are stated, are provided here.

The original Figure 2 appears below as Figure 1.

**Figure 1:**
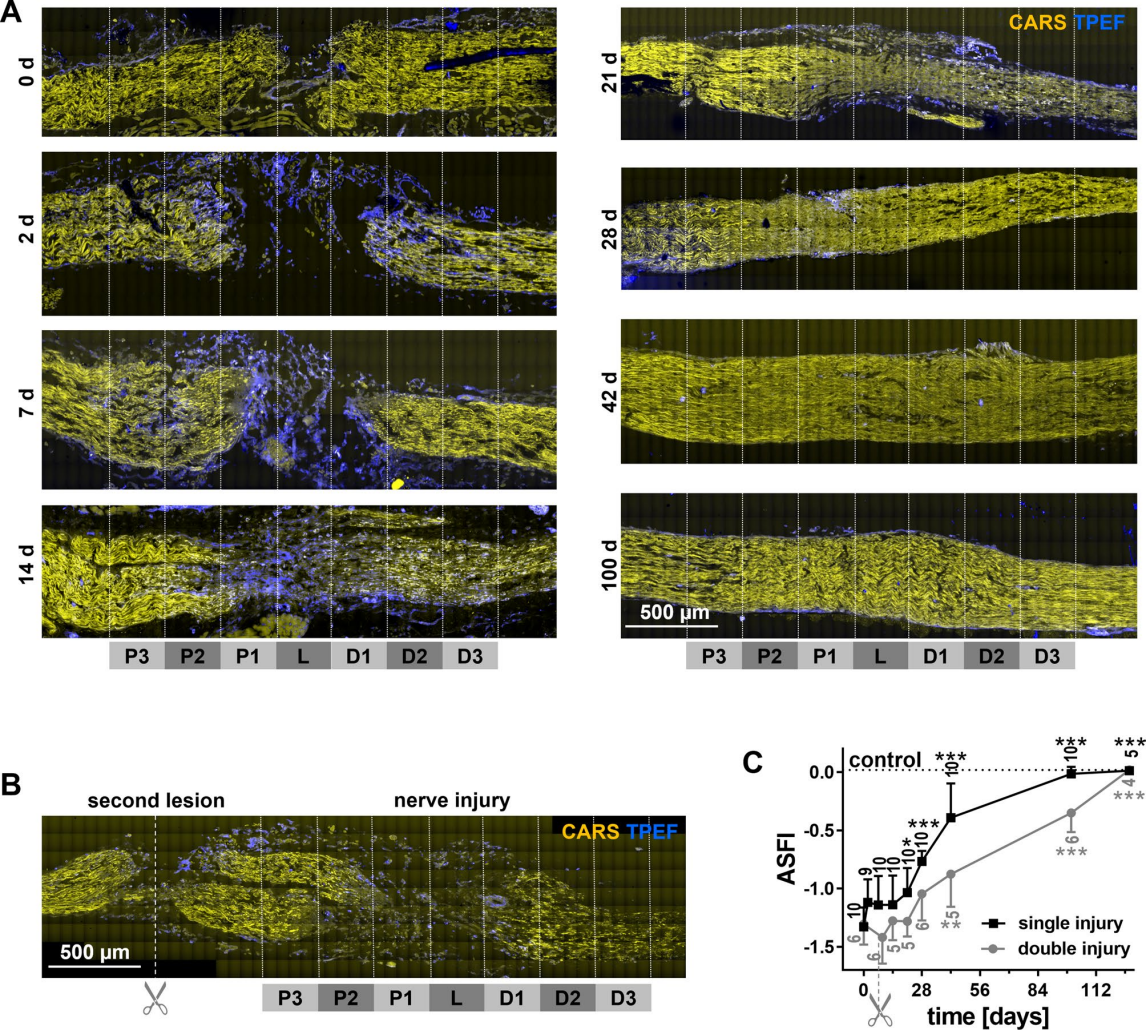
Label-free multiphoton microscopy of the axolotl sciatic nerve and functional outcome after injury. (**A**) CARS/TPEF-images of sciatic nerve longitudinal sections at different time points after nerve injury (single injury model) (**B**) CARS/TPEF image of the sciatic nerve nine days after injury and two days after second lesioning (double injury model). The regions for quantitative analysis are indicated in A and B: P3-1: proximal 3-1; L: lesion; D1-3: distal 1-3. (**C**) Axolotl sciatic nerve functional index (ASFI) for both injury models. Dotted line indicates ASFI of animals without injury (control). Asterisks indicate significant differences to the ASFI at 0d, i.e. directly after injury (*P < 0.05, **P < 0.01, ***P < 0.001; one way ANOVA followed by Bonferroni’s multiple comparisons test).

The original Figure 3 appears below as Figure 2.

**Figure 2:**
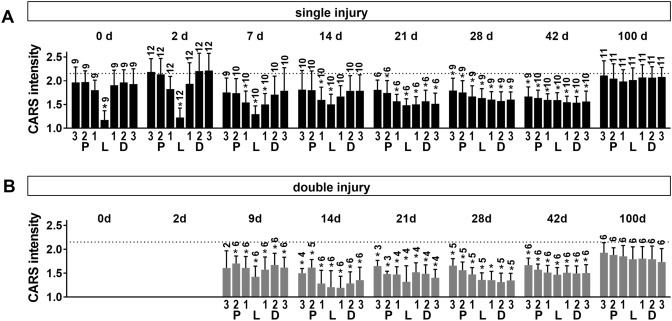
CARS signal intensity after sciatic nerve injury. Normalized CARS signal intensity in regions proximal to the lesion (P 3-1), distal to the lesion (D 1-3) and within the lesion (L) as indicated in Fig. 2A/B at different time points after initial injury of the sciatic nerve. (**A**) single injury model (**B**) double injury model. Bars show mean and SD. Dotted line indicates CARS intensity of intact control nerve. Asterisks indicate significant differences vs. control (P < 0.05, one way ANOVA followed by Bonferroni’s multiple comparisons test).

The original Figure 4 appears below as Figure 3.

**Figure 3:**
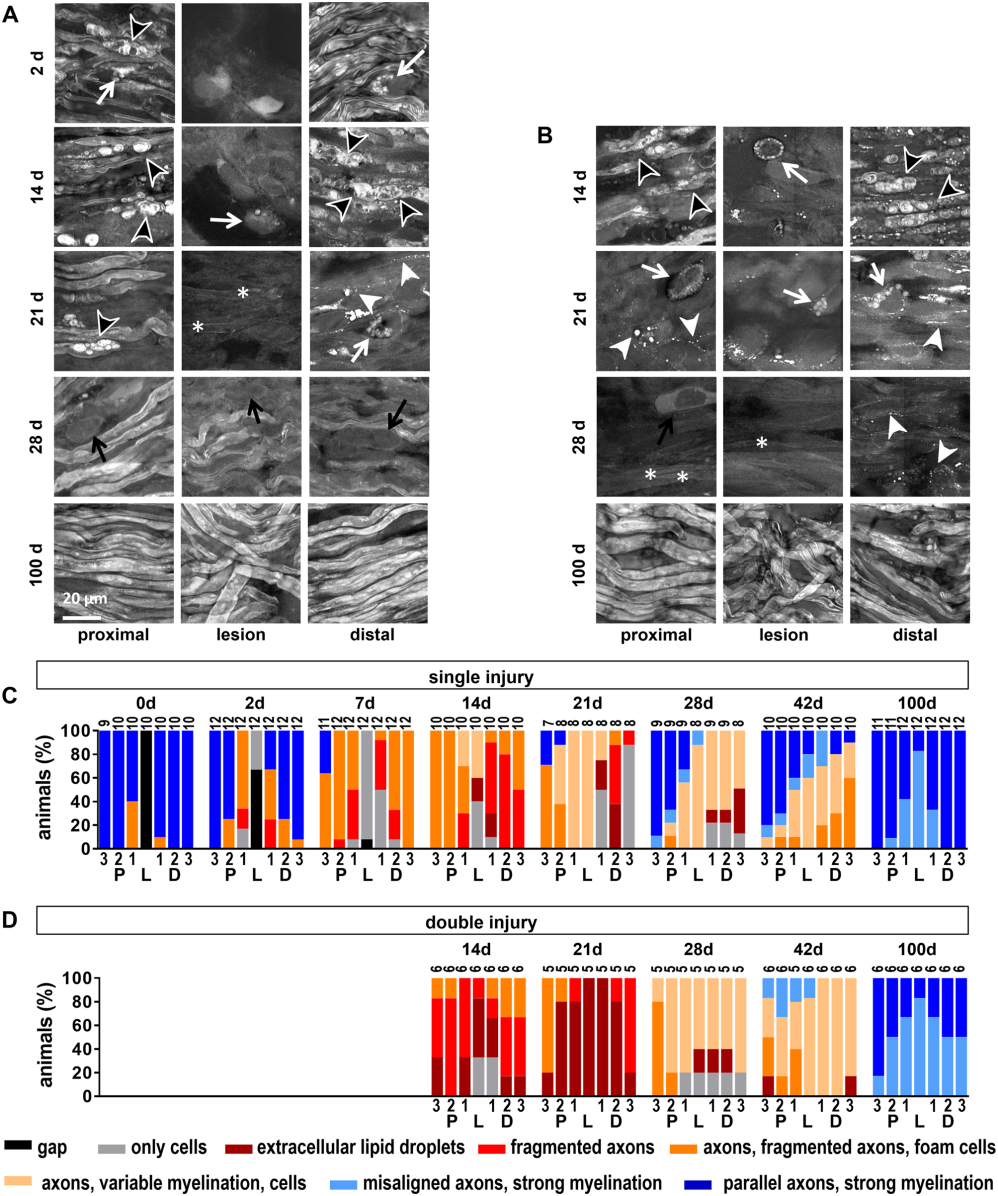
Micromorphology of the regenerating nerve shown by CARS. (**A**) CARS images at different time points after sciatic nerve dissection as indicated for single injury model. Representative examples proximal to the lesion, at the lesion and distal to the lesion. (**B**) Reference immunohistochemistry for neurofilament H of the axolotl sciatic nerve 21 d after injury. The region of the lesion is indicated (L) (**C**) CARS images at different time points after sciatic nerve dissection as indicated for double injury model. White arrows: Cells with intracellular lipid droplets, black arrows: Cells, white arrowheads: Extracellular lipid droplets, black arrowheads: Ovoids, asterisk: Not/weakly myelinated axons. (**D/E**) Semi quantitative analysis of the micromorphology based on CARS imaging in the single (**D**) and double (**E**) injury model using a scoring system. Quantification was performed in seven regions along the nerve (P3-1, L, D1-3, indicated in Fig. 2A/B). The bars indicate the percentage of samples that fall in the given categories.

The original Figure 6 appears below as Figure 4.

**Figure 4:**
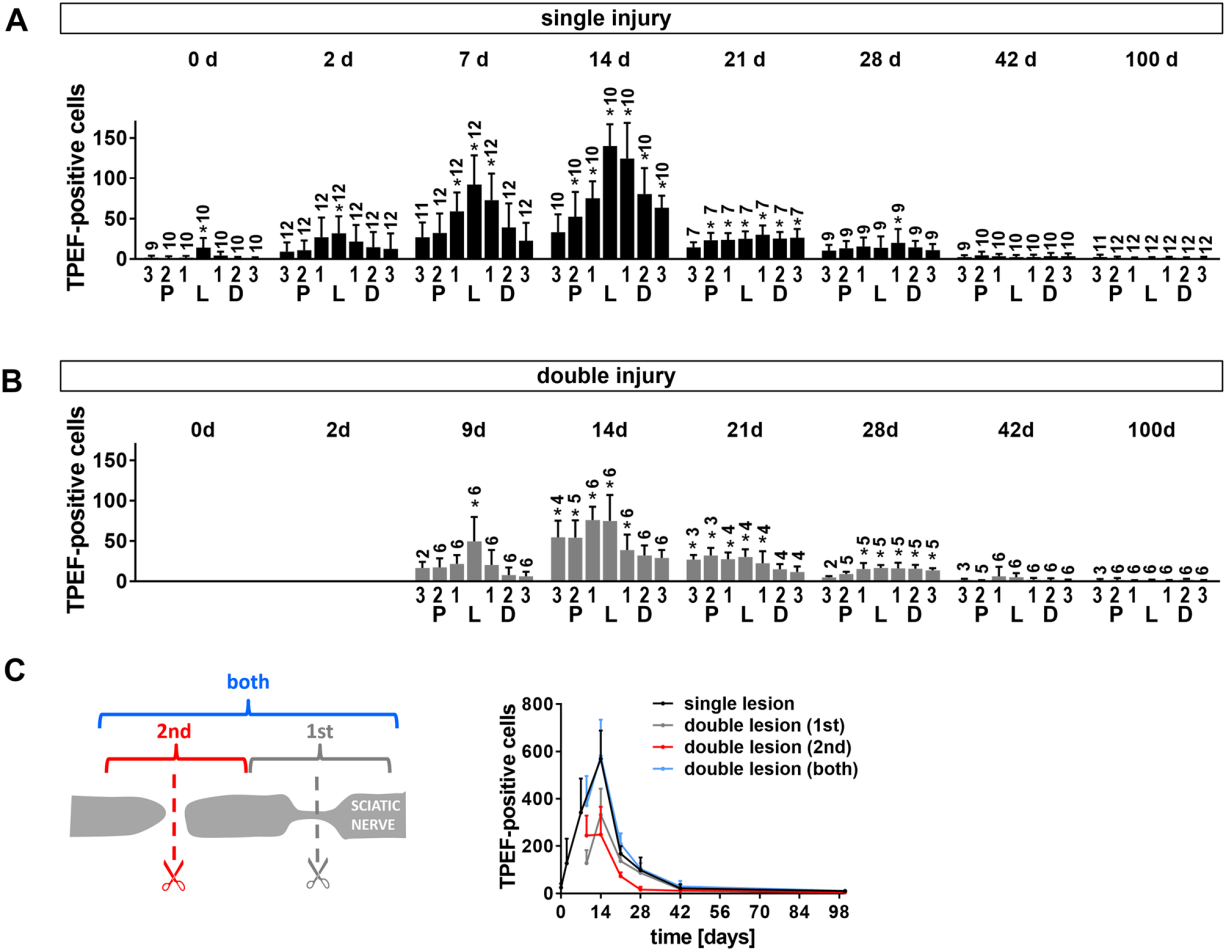
Changes in TPEF-positive cells after sciatic nerve injury. (**A**) Number of TPEF-positive cells in regions proximal to the lesion (P3, P2, P1), distal to the lesion (D1, D2, D3) and within the lesion (L) at different time points after initial injury of the sciatic nerve. (**A**) single injury model (**B**) double injury model. Bars show mean and SD. Asterisks indicate significant differences vs. control (intact nerve). (**C**) Total number of TPEF positive cells (sum of all regions investigated) at different time points after nerve transection (mean and SD).

