# Supplementary material for: Label-free Imaging of Tissue Architecture during Axolotl Peripheral Nerve Regeneration in Comparison to Functional Recovery
Source: Sci Rep. 2019 Sep 2;9:12641. doi: 10.1038/s41598-019-49067-3 (PMC6718386; doi:10.1038/s41598-019-49067-3)
Supplement: Supplementary file 1 — Supplemantary Material [file 41598_2019_49067_MOESM1_ESM.pdf]

Supplementary Materials:

Label-free Imaging of Tissue Architecture during Axolotl Peripheral Nerve Regeneration in Comparison to Functional Recovery

Authors: Ortrud Uckermann[1]†*, Joana Hirsch[1]†, Roberta Galli[2], Jonas Bendig[1], Robert Later[1,3] Edmund Koch[2,3], Gabriele Schackert[1], Gerald Steiner[2], Elly Tanaka[3],Matthias Kirsch[1,3]

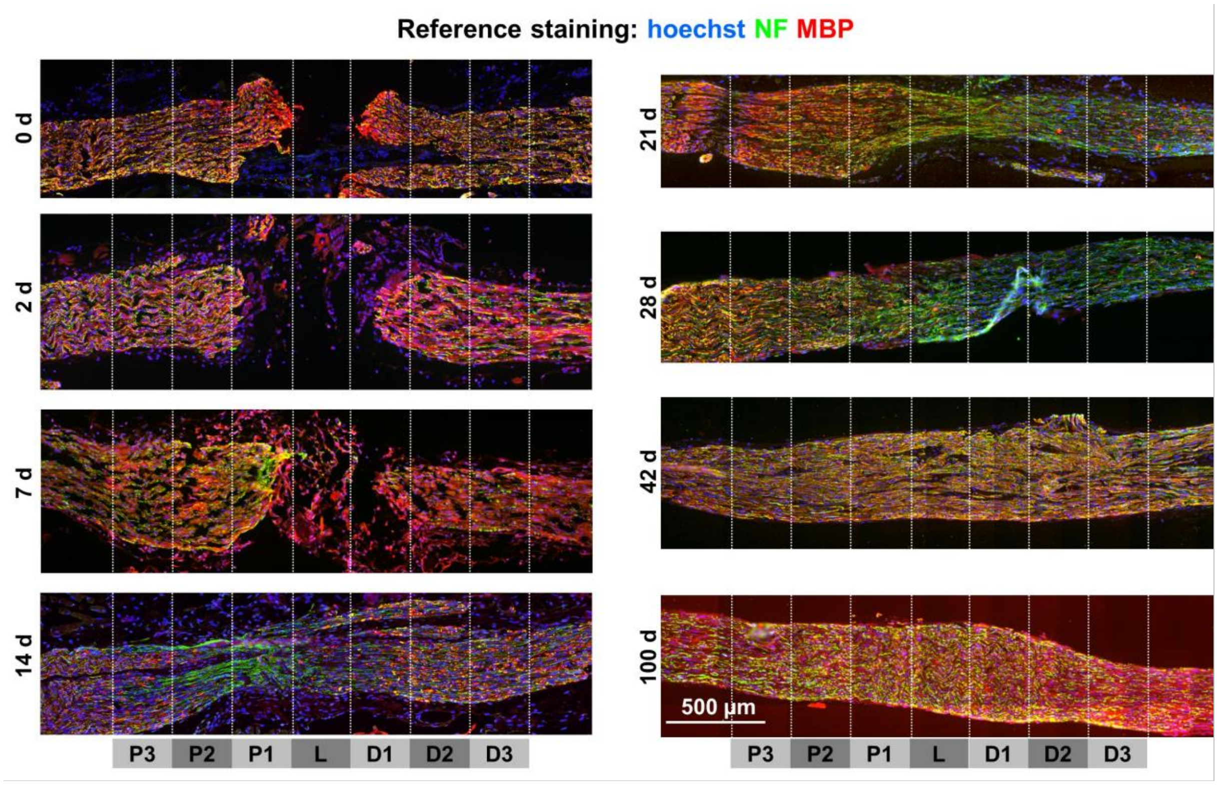

Supplementary Figure S1. Reference immunohistochemistry for neurofilament and myelin of the axolotl sciatic nerve after injury. Neurofilament H (green) and myelin basic protein (red) immunohistochemistry of the same or consecutive section as shown in Figure 1. Nuclei are stained by Hoechst (blue) The time points after nerve injury and regions used for quantitative analysis of label free CARS/TPEF imaging are indicated: P3-1: proximal 3-1; L: lesion; D1-3: distal 1-3.

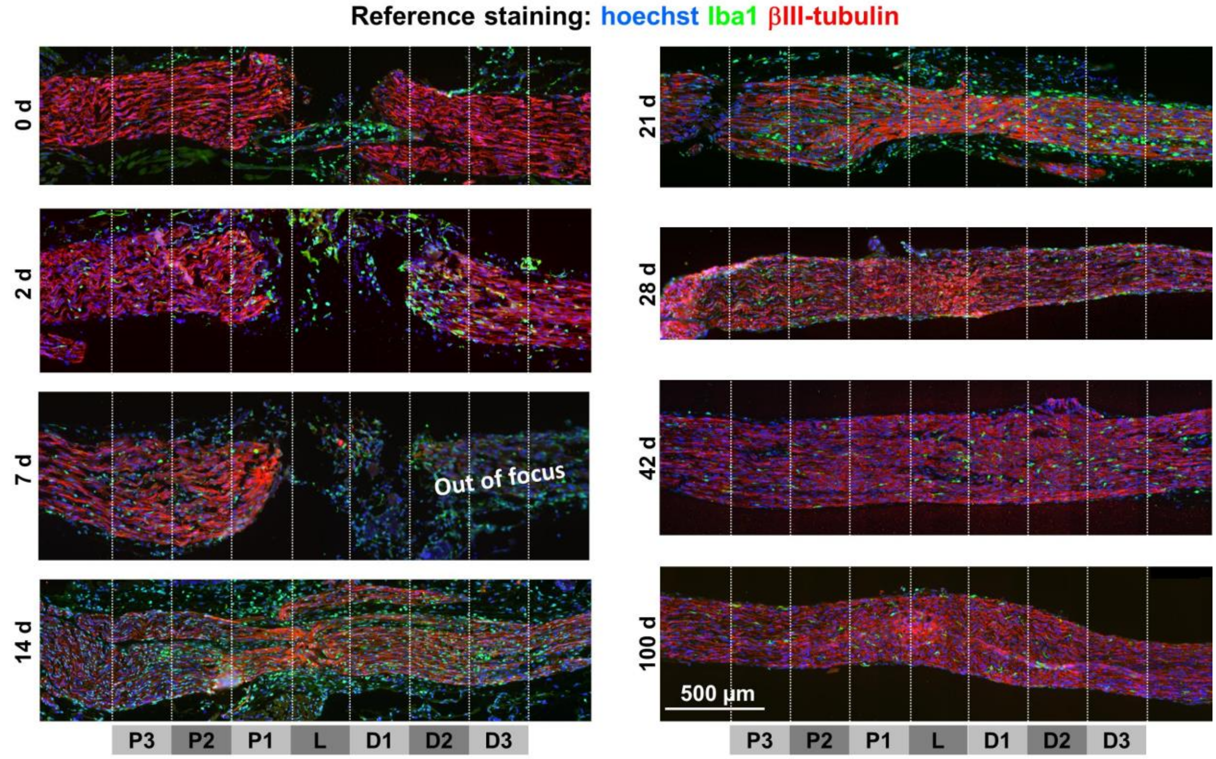

Supplemental Figure S2. Reference immunohistochemistry for microglia/macrophages and neuronal marker of the axolotl sciatic nerve after injury. Iba1 (green) and βIII-tubulin (red) immunohistochemistry of the same or consecutive section as shown in Figure 1. Nuclei are stained by Hoechst (blue) The time points after nerve injury and regions used for quantitative analysis of label free CARS/TPEF imaging are indicated: P3-1: proximal 3-1; L: lesion; D1-3: distal 1-3.
